# Efficacy of Low-level Laser Therapy, Hyaluronic Acid Gel, and Herbal Gel as Adjunctive Tools in Gingivectomy Wound Healing: A Randomized Comparative Clinical and Histological Study

**DOI:** 10.7759/cureus.6438

**Published:** 2019-12-21

**Authors:** Sana Priyanka Reddy, Rekha R Koduganti, Veerendranath Reddy Panthula, Jammula Surya Prasanna, Himabindu Gireddy, Rajashree Dasari, Manasa Ambati, Bharath Chandra G

**Affiliations:** 1 Periodontics, Panineeya Mahavidyalaya Institute of Dental Sciences and Research Centre, Hyderabad, IND

**Keywords:** gingivectomy, lllt, gengigel, hiora sg gel, polarized microscopy

## Abstract

Introduction

Gingival overgrowth is usually an inflammatory response to plaque present on tooth surfaces. The other causes could be drugs and other systemic conditions. When the local factors are responsible and subgingival scaling does not help, gingivectomy is performed. The gingivectomy wound is raw and heals slowly. Low-level laser therapy (LLLT), hyaluronic acid, and herbal gels aid in healing after a gingivectomy. This study compared the efficacy of LLLT, hyaluronic acid, and herbal gel when used topically after a gingivectomy. This was a single-arm, interventional trial wherein 30 patients aged between 18 and 40 years with moderate gingival overgrowth participated. The study was approved by the institutional ethical committee. (DN/0109-16). The participants signed the consent form and the study was also registered (NCT03569683).

Materials and methods

The samples were equally divided into three groups. Group A received LLLT immediately postop, and on the first, third, and seventh days after surgery. Group B received hyaluronic acid (Gengigel) while Group C received an herbal gel (Hiora SG) for the same time periods after surgery, respectively. Analysis of variance (ANOVA) followed by a post-hoc Bonferroni test was used to evaluate differences within groups. Intergroup comparison was performed using the independent t-test. A p-value of <0.05 was considered statistically significant.

Results

The plaque index (PI), gingival index (GI), and gingival enlargement index (GEI) showed good improvement postoperatively within the groups, which was statistically significant. However, on an intergroup comparison, the GEI pertaining only to the laser group showed significant changes. Also, the pain perception analyzed by the visual analog score (VAS) was the least, and histologically, the amount of mature collagen fibers laid down were more in the laser group.

Conclusion

Patients irradiated with laser after gingivectomy (Group A) showed superior results in the clinical, histological variables as compared to Groups B and C.

## Introduction

Gingival enlargement (hyperplasia) is a condition often occurring due to plaque being present on the tooth surface. Systemic intake of drugs and some systemic conditions can exacerbate the same. If the overgrowth is due to local factors, the removal of the same by subgingival scaling will cause the regression of the overgrowth. But sometimes, the enlargement remains even after repeated scaling and root planing. In such instances, the gingiva has to be excised surgically. The wound produced by gingivectomy heals by secondary intention. It takes six weeks for the epithelization to be completed. During this period, patients experience a lot of pain and discomfort. To alleviate this discomfort, topical agents like zinc have been employed in the past. This study was done to assess the efficacy of low-level laser therapy (LLLT), hyaluronic acid gel, and herbal gel when used topically after a gingivectomy.

LLLT augments the healing of soft tissues by increasing the metabolic activity of cells. There is an increased proliferation of epithelial cells and fibroblasts and an increase in the synthesis of the extracellular matrix. It has indicated a good response in the repair of open wounds like burns and ulcerative lesions [1-2].​^ ^The^ ^hyaluronic acid gel has anti-inflammatory properties and has been observed to induce more epithelial tissue formation, as well as an increase in vascular supply to the connective tissue [3-4]. The herbal gel has astringent and anti-inflammatory properties [5].

## Materials and methods

The gingival enlargement was graded according to the index by Miller and Damn [6]. Patients with suprabony pockets and with gingival enlargement index (GEI) ≥2 mm participated in the study. Smokers, diabetic patients and those having infrabony pockets were excluded. All the patients underwent the preliminary phase wherein scaling and root planing, occlusal adjustments, and dietary evaluation were done.

A total of 30 patients of both sexes with moderate (>2 mm) gingival overgrowth were selected from the outpatient ward of a hospital in Hyderabad and equally distributed into three sections. To achieve a mean difference of 1.94 between the groups with the level of significance being 0.05 and power 95%, it was assessed that 10 patients per group would be sufficient.

The samples were screened and randomly assigned in sealed envelopes by investigator KRR into LLLT (Group A), hyaluronic acid gel (Group B), and herbal gel (Group C). The treatment was performed by investigator SP.

The site earmarked for surgery was anesthetized with 2% lignocaine (1 in 80,000). A Williams probe was used to verify the pocket depth, which was marked using a pocket marker. External bevel gingivectomy using BP blade no. 15 was initiated. The incision was made 1 mm apical to the pockets marked. Then, the tissue was excised using periodontal knives. After the removal of the excised pocket wall, the area was cleared of granulation tissue using curettes and shaped to prevent the formation of ledges, which favor plaque accumulation. Saline was used to irrigate the operated site. After the gingivectomy, the patients were treated either with LLLT (Group A), hyaluronic acid gel (Group B), or herbal gel (Group C).

**Figure 1:**
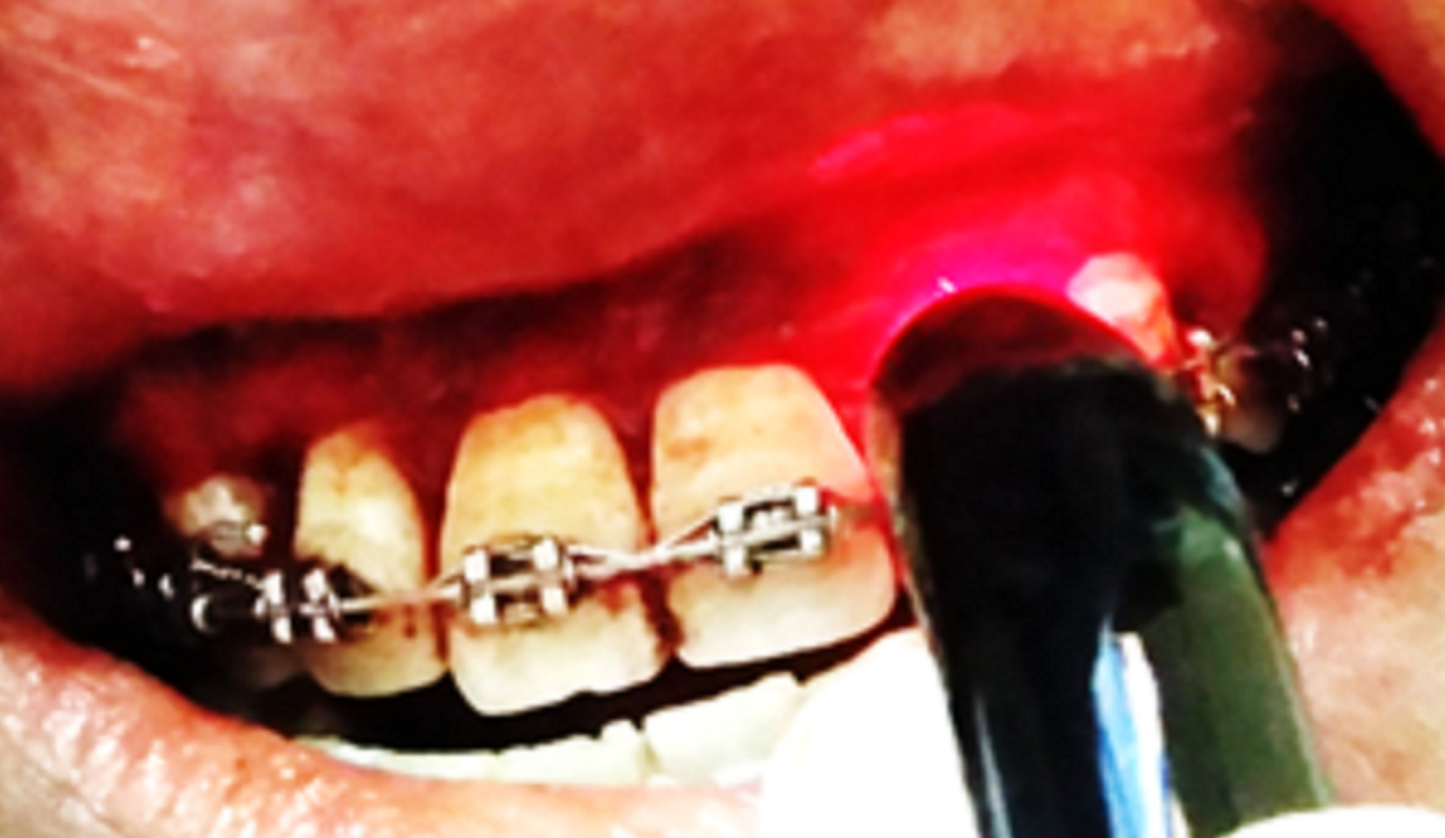
Laser biostimulation

In Group A, diode laser biostimulation (using a biostimulation probe), 980 nm, an output power of 50 mW, and an energy density of 4J/cm on the surgical site was carried out in the contact defocussed mode for three minutes and repeated on the first, third, and seventh days postsurgery (Figure 1).

**Figure 2:**
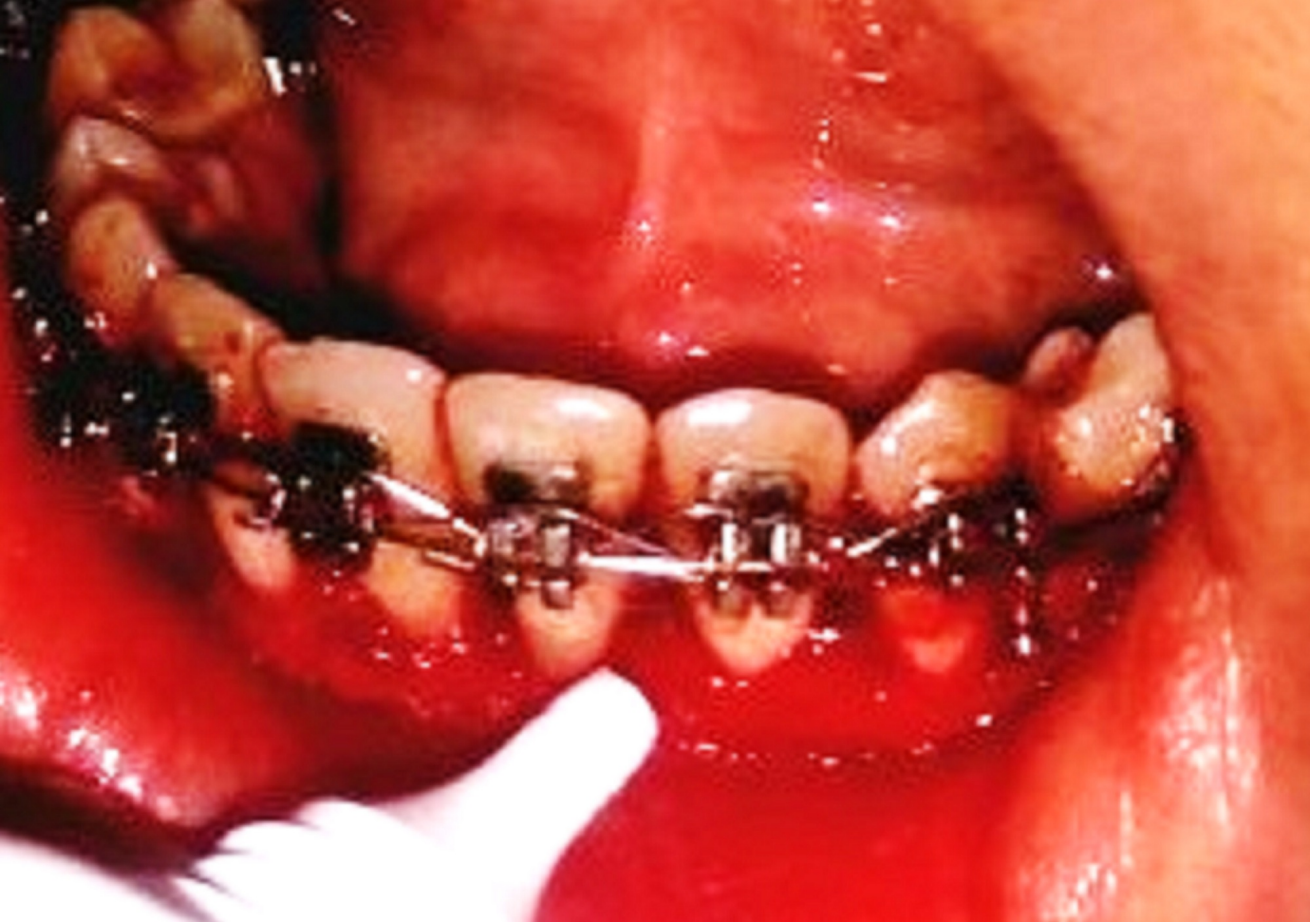
Hyaluronic acid gel topical application

In Group B, hyaluronic acid gel (Ricerfarma, Milano, Italy) was applied topically on the surgical site. Then, a tin foil was placed over which a periodontal dressing was given and this was repeated on the earlier mentioned days after surgery (Gengigel formulation: 0.2% hyaluronic acid, and 7.5% xylitol). Hyaluronic acid has good anti-inflammatory and wound-healing properties. As there are no studies wherein hyaluronic acid (Gengigel) had been applied topically after gingivectomy, this study included Gengigel to assess its wound-healing properties (Figure 2).

**Figure 3:**
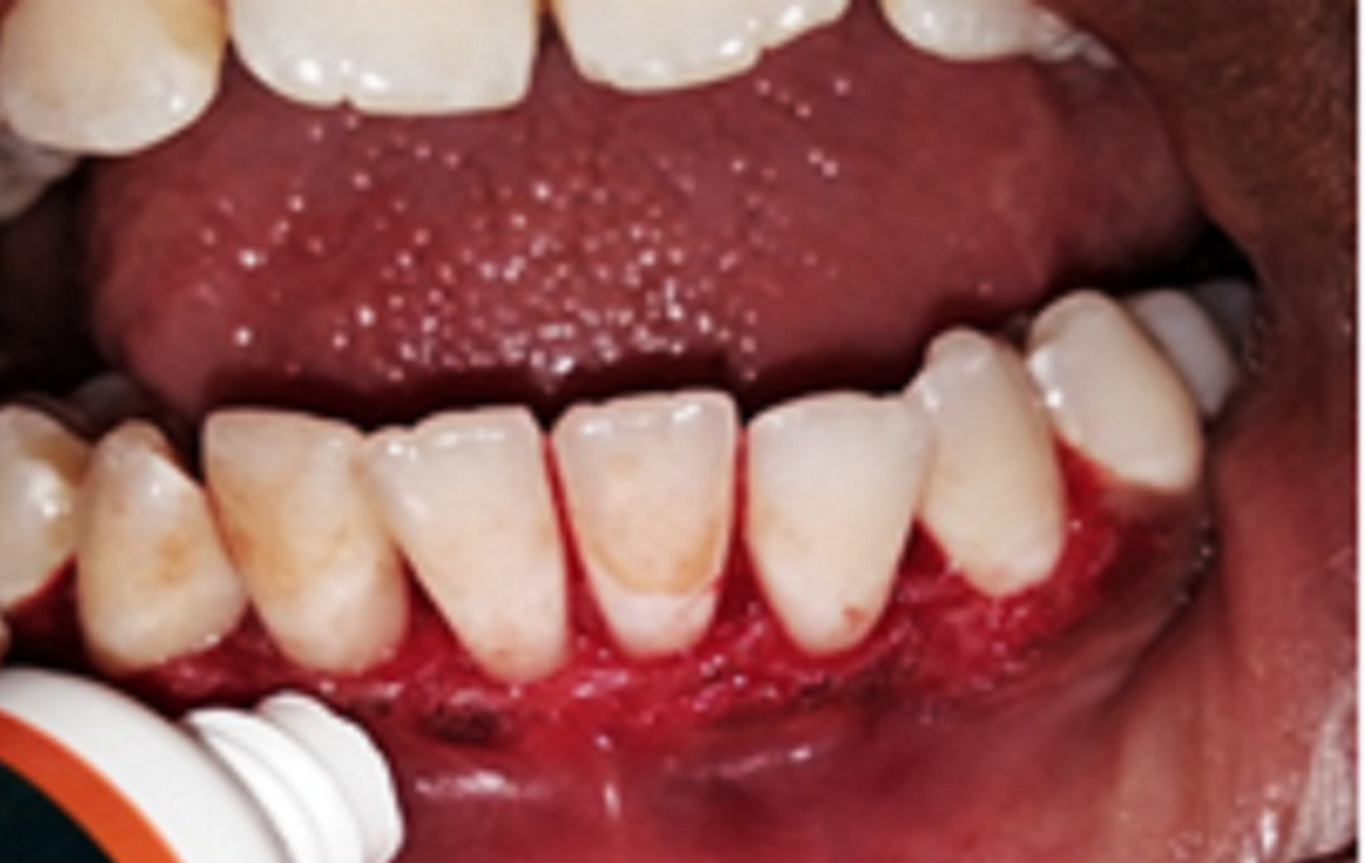
Herbal gel topical application

In Group C, a herbal gel was applied topically on the surgical site, then a tin foil was placed over which a periodontal dressing was given and was repeated, on the same days as mentioned for the other two groups post-surgery. Hiora SG gel (The Himalaya Drug Company, Makali, Bengaluru) contains: jasmine (jati), licorice (yashtimadhu), spreading hogweed (punarnava), and triphala (Figure 3). Since herbal products have minimal side effects and as there no studies reported using herbal topical agents after gingivectomy, Hiora SG Gel was included in the study.

All the patients received appropriate antibiotics (amoxicillin 500 mg thrice daily for five days) and analgesics (aceclofenac 100 mg two times a day for five days). The periodontal dressing was removed after one week. Patients were given instructions to brush with care and were reinstructed about oral hygiene maintenance. They were examined at weekly intervals until six weeks after surgery.

The PI, GI, and GEI were assessed at baseline and six weeks post-surgery. Clinical evaluation was also done after seven days by using a 10-point visual analog scale (VAS) to score the healing of the surgical wounds. VAS consists of a horizontal line of 10 cm with two end-points representing 'no pain' and 'worst pain.’ Patients were asked to mark a point on the line to show their magnitude of pain (0: No pain 1-3: Slight pain 3.1-6: Moderate pain 6.1-10: Severe pain).

The histological evaluation was done using a polarized microscope, using Picrosirus staining [7] six weeks post-gingivectomy to assess the quality and quantity of collagen fibers laid down. The Picrosirius red stain (also called "Sirius red" stain) is one of the best-understood histochemical techniques able to selectively highlight collagen networks. The technique relies on the birefringent properties of collagen molecules. While the picrosirius red stain alone does not selectively bind the collagen network, it becomes more specific than the other common collagen stains when combined with polarized light detection. The stained sections showing stromal collagen fibers in the three experimental groups were captured with a camera with a 40x objective. Fiber thickness was determined on the image captured, using image analysis software (ImageJ) [8]. All the measurements were assessed in micrometers.

Care was taken in procuring 0.5 mm of tissue from the interdental papilla between the canine and first premolar, which was processed for examination using Picrosirius staining. This was performed six weeks postop to record the new collagen laid down. The reagents used were Sirius red dye (Sigma-Aldrich, St. Louis, Missouri, United States), picric acid, xylene, DPX mounting media. "The procedure was done by initially dewaxing and hydrating the paraffin sections. Then, the nuclei were stained with Mayer’s hematoxylin for 8 minutes and then the slides were washed for 10 minutes in running tap water. The slides were then stained with Picrosirius red for one hour. They were then washed in two changes of acidified water and the water from the slides was removed by vigorous shaking. Later, the slides were dehydrated in three changes of 100% ethanol. They were then cleared in xylene and mounted in a resinous medium. 5μm sections were made and stained with Picrosirius red stain and observed under a polarized microscope."

## Results

The red-stained sections depicting stromal collagen fibers in the three groups were captured with a camera with a 40x objective. Fiber thickness was determined on the image captured using image analysis software (ImageJ). All the measurements were in micrometers. In each case, three high-power fields (40x) were selected and from each field, the thickness of fibers was measured. The thickness of the fibers was ranging from 0.1-2.4 micrometers. Based on thickness, the collagen fibers were divided into thin and thick fibers and the number of fibers was calculated. Thin fibers ranged from 0.1-0.80 micrometers. Thick fibers 0.81-2.4 micrometers. The data recorded was marked on an Excel sheet for further interpretations and statistical analysis. (Figures 5-7).

**Figure 4:**
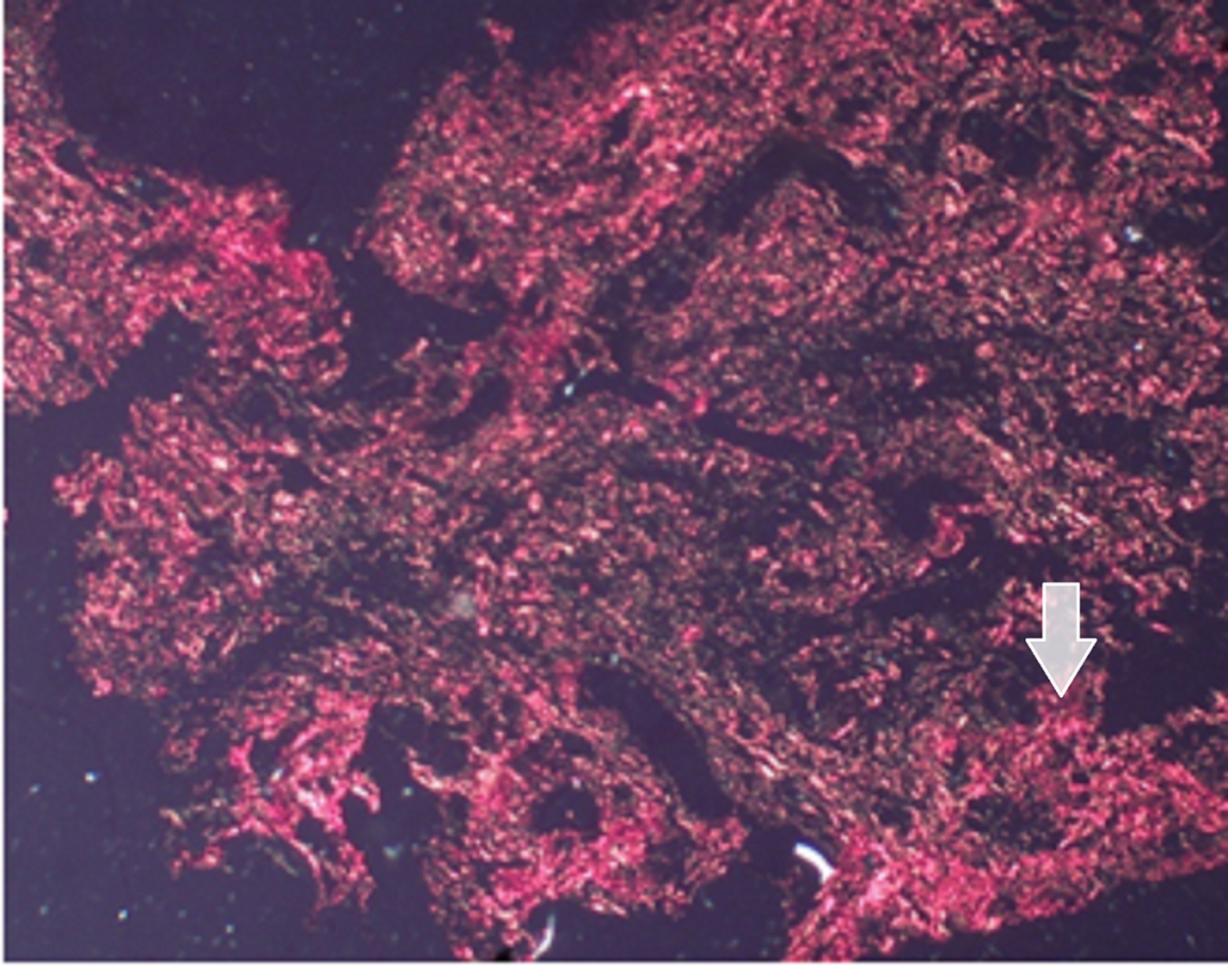
Laser group (Group A) showing OR birefringence of thick collagen fibers 40X Magnification

**Figure 5:**
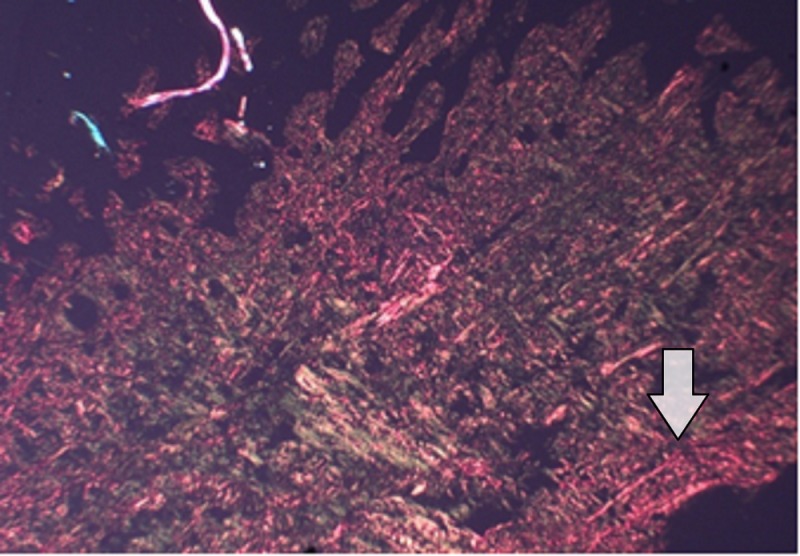
Image of hyaluronic acid gel group (Group B) showing moderately well-formed collagen fibers 40X Magnification

**Figure 6:**
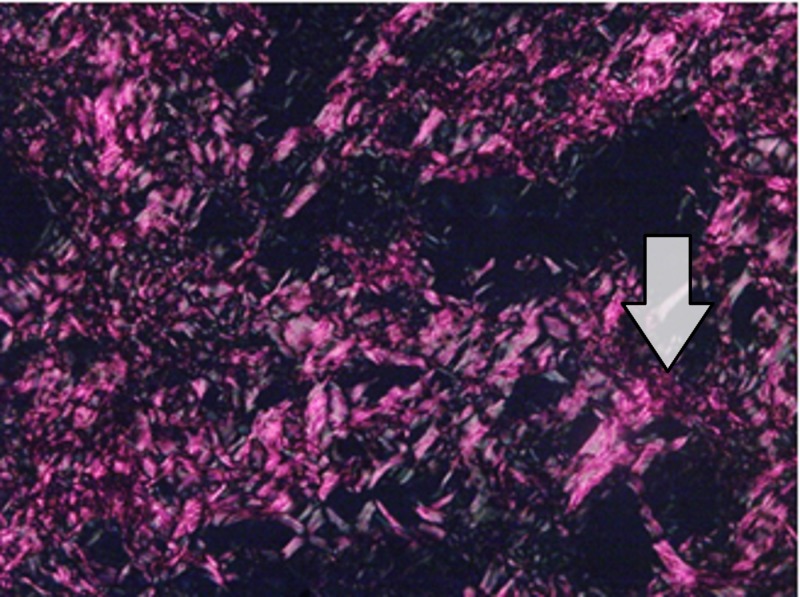
Image of the herbal gel group (Group C) showing thin collagen fibers 40X Magnification

The reduction in PI, GI, and GEI six weeks postoperatively was statistically significant when compared with baseline in all three groups (Table 1).

**Table 1:** Intragroup comparison among groups using the paired t-test LLLT: low-level laser therapy Hiora SG: The Himalaya Drug Company, Makali, Bengaluru

Group	Variables	Baseline		Post (6 Weeks)		Difference		P-Value	Significant
LLLT (Group A)	PI	1.64	0.32042	0.99	0.3755	0.65	0.1118	0	SIG
	GI	2.06	0.189	1.03	0.539	1.03	0.43729	0	SIG
	GEI	2.3	0.483	1	0	1.3	0.483	0*	SIG
Gengigel (Group B)	PI	1.585	0.334	1	0.456	0.585	0.41836	0.002*	SIG
	GI	2.38	0.456	1.4	0.581	0.98	0.18738	0*	SIG
	GEI	2.3	0.483	1.1	0.316	1.2	0.421	0*	SIG
Hiora SG gel (Group C)	PI	1.7	0.21602	1.05	0.57591	0.65	0.55025	0*	SIG
	GI	2.39	0.4508	1.46	0.769	0.93	0.67831	0.002*	SIG
	GEI	2.3	0.483	1.4	0.516	0.9	0.316	0*	SIG

When the values were compared between the three groups, there was an improvement in the values of GEI only (P<0.05). The GEI at baseline was 2.3±0.483 and six weeks postop was 1±0 in the LLLT group. The baseline values of GEI for the Gengigel group were 2.3±0.483, which reduced to 1.1±0.316, six weeks after the procedure. The Hiora SG group recorded a GEI of 2.3±0.483 before surgery, which improved to 1.4±0.516, 6 weeks later (P<0.05) (Table 2).

**Table 2:** Intergroup comparison among groups using ANOVA ANOVA: analysis of variance; GEI: gingival enlargement index; GI: gingival index Hiora SG: The Himalaya Drug Company, Makali, Bengaluru

Clinical Parameters	Laser (GROUP A)		Gengigel (GROUP B)			Hiora SG Gel (GROUP C)		P-Value	Sig
	Mean	SD	Mean	SD		Mean	SD		
PI	1.64	0.32042	1.585		0.334	1.7	0.21602	0.687	NS
PI_POST	0.99	0.3755	1		0.456	1.05	0.57591	0.956	NS
GI	2.06	0.189	2.38		0.456	2.39	0.4508	0.113	NS
GI_POST	1.03	0.539	1.4		0.581	1.46	0.769	0.28	NS
GEI	2.3	0.483	2.3		0.483	2.3	0.483	0.165	NS
GEI_POST	1	0	1.1		0.316	1.4	0.516	0.043*	SIG

Related to the VAS between the groups at one week postsurgery, it was observed that the score was the least in the LLLT group, followed by the Gengigel group and the Hiora SG gel group (Figure 8).

**Figure 7:**
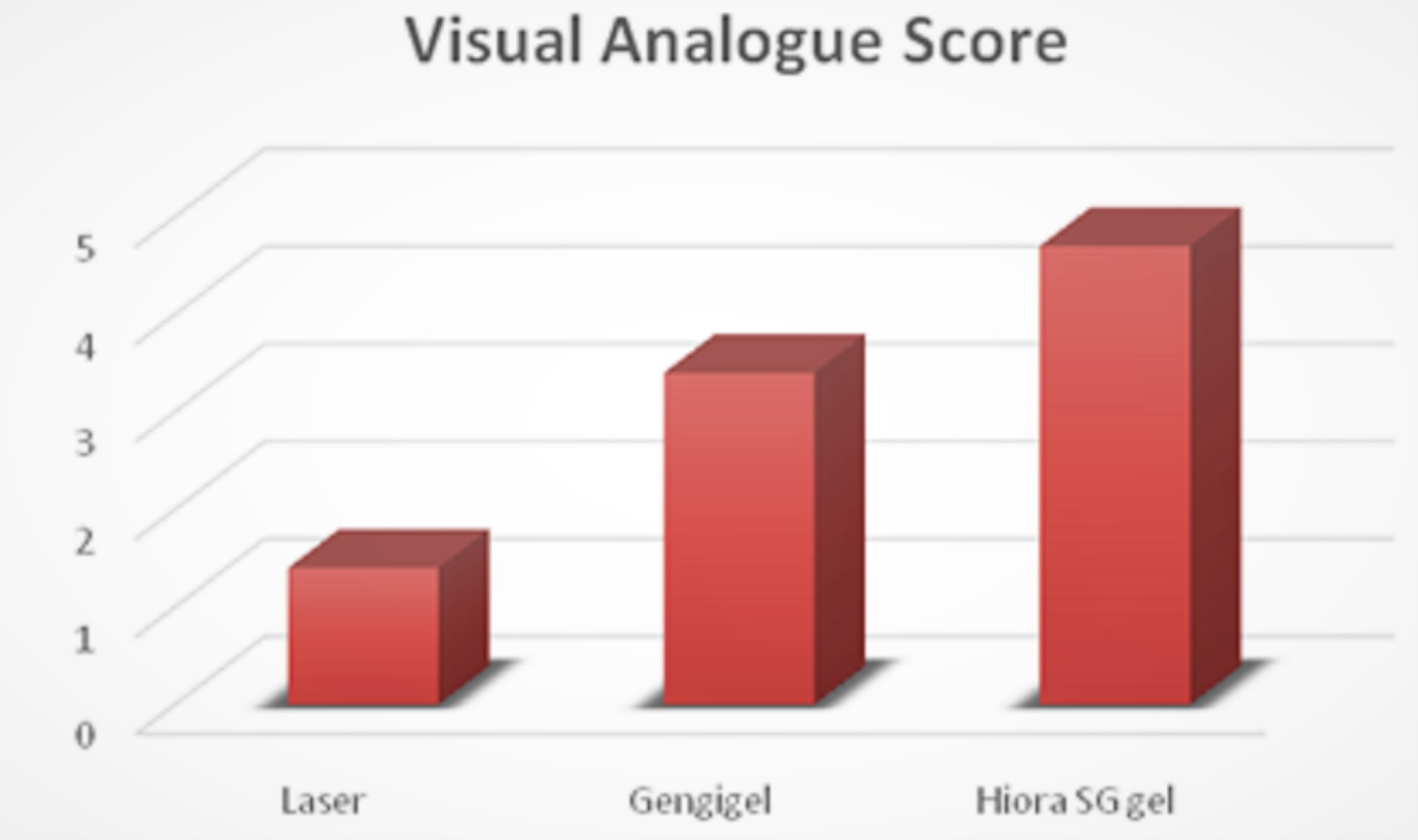
Comparison of postoperative visual analog scores between the groups

Pertaining to the histological evaluation, the mean values of thin green-yellow (GY) fibers among groups were not statistically significant. The mean values of thin orange-red (OR) fibers were 34.7±8.49 for the LLLT group, 22.36±5.20 for the Gengigel group, 18.33±3.62 for the Hiora SG gel group and were statistically significant. The mean value of thick GY fibers was 16.06±2.15 in the LLLT group, 14.56±1.98 in the Gengigel group, and 17.7±1.95 in the Hiora SG gel group and was statistically significant. Also, the mean values of thick OR fibers were 31.1±6.27 for the LLLT group, 22.8±5.06 for the Gengigel group, and 20.5±4.11 for the Hiora SG group, which was statistically significant. On an intergroup comparison, a difference in thick GY, thick OR, and thin OR fibers was noted in Group A. Hence, it can be inferred that the LLLT group has shown a predominance of collagen fibers with orange-red birefringence, indicating the presence of mature collagen as compared to the Gengigel and Hiora SG Gel groups (Table 3).

**Table 3:** Intergroup comparison between thick and thin fibers using the ANOVA test ANOVA: analysis of variance

Collagen Fibers	Group						P-value	SIG
	Laser (GROUP A)		Gengigel (GROUP B)		Hiora SG Gel (GROUP C)			
	Mean	SD	Mean	SD	Mean	SD		
THIN GY	17.233	1.678	16.567	2.554	16.567	2.63	0.763	NS
THIN OR	34.732	8.497	22.366	5.2048	18.332	3.627	0^*^	SIG
THICK GY	16.068	2.157	14.567	1.982	17.7	1.951	0.007*	SIG
THICK OR	31.1	6.278	22.831	5.069	20.534	4.116	0*	SIG

## Discussion

Gingival enlargement is often encountered by clinicians and its apt treatment depends on detecting the etiopathogenesis [9]. The microbe-laden plaque plays a pivotal role in inflammatory gingival enlargement. There are different therapeutic modalities to eliminate periodontal pockets, depending on the type and relation to the alveolar crest. When the pockets are supra-alveolar, they are called pseudo-pockets, as there is no loss of attachment but a coronal enlargement of the gingival margin. Poor oral hygiene and trauma to the gingiva by improper restorations and orthodontic appliances are the main reasons [10].

The selection of an appropriate therapeutic technique depends on the state, as well as the size of the gingival overgrowth. When there is no regression in the size of the gingiva, even after repeated scaling and root planing, gingivectomy is done. There are different techniques for performing gingivectomy: conventional, laser, electrocautery, and cryosurgery. Among these techniques, conventional gingivectomy is considered the gold standard [11].

In a study comparing conventional gingivectomy with the laser protocol, researchers observed that both the plaque index and the gingival index reduced postoperatively in both the groups when the pockets were <3 mm; however, in deeper pockets, conventional gingivectomy yielded better results [12].

Gingivectomy wounds heal by secondary intention. It takes approximately four weeks for the healing to occur clinically and six weeks for it to be completed histologically. To hasten the healing process, various agents have been experimented upon and in this study, we have compared the healing between LLLT, hyaluronic acid gel, and herbal gel.

LLLT applied to soft tissues excites specific metabolic processes in healing wounds. The major changes observed include increased granulation tissue, early epithelialization, increased fibroblast proliferation, and matrix synthesis. Laser use requires minimal anesthesia and less operative time and results in good postoperative healing.

Researchers in a study assessing gingival healing after gingivectomy, with the use of adjunctive LLLT, observed that a 685-nm wavelength laser with an output power of 50 mW and an energy density of 4 J/cm was effective as an adjunct in promoting healing [13].

In another study, the authors evaluated the effects of LLLT after gingivectomy and gingivoplasty. The researchers observed that LLLT using 588 nm wavelength, accelerated epithelization, and wound healing [14].

In this study also it was observed that there was a reduction in PI (1.64±0.99), GI (2.06±1.03), and GEI (2.3±1.0) from preop to postop in the laser group using a wavelength of 980 nm, output power of 50mW, and energy of 4J/cm (Group A) (Table 1).

Hyaluronan present in the extracellular matrix of all vertebrates is a glycosaminoglycan playing a pivotal role in scarless wound healing. It aids in the early deposition of healthy granulation tissue by promoting epithelial cell turnover, good vascularity to the connective tissue, and inhibiting inflammation. When topically applied hyaluronic acid has been observed to hasten healing in patients with gingivitis and periodontitis [3].

A study showed that when 0.2% HA was applied topically, twice daily for a three-week period, it had a beneficial effect in patients with gingivitis. There was an improvement in the plaque index, papillary bleeding index (PBI), and gingival crevicular fluid (GCF) variables [4].

In another study, 0.2% HA gel, when applied on inflamed gingiva, twice daily for a four-week period, as an adjunct to SRP, showed a marked reduction in the gingival index (GI) and PBI when compared with both the control group (scaling plus placebo gel) and the negative control group (scaling only) [15].

In the present study, there was an improvement in PI (1.585±1.0), GI (2.06±1.03), and GEI (2.3±1.1) six weeks postoperatively in the Gengigel group (Group B) (Table 1).

The Hiora SG gel is an herbal gel with analgesic, anti-inflammatory, and antibacterial properties. It repairs the damaged oral mucosa and has often been used to treat aphthous ulcers. In a study evaluating the efficacy of Hiora SG gel in patients with stomatitis, it was observed that the gel, significantly reduced the swelling, pain, and size of the ulcer at the end of three weeks of treatment. It was thus concluded that Hiora SG gel was clinically effective and safe in the management of stomatitis [5].

As per our knowledge, Hiora SG gel has not been used as a topical medicament after gingivectomy, hence, it was decided to evaluate the effects of this herbal gel. In the present study, there was a marked improvement in PI (1.7±1.05), GI (2.39±1.46), and GEI (2.3±1.4) in the Hiora SG (Group C), six weeks after the gingivectomy (Group C) (Table 1).

Though all the three test groups showed an improvement in the clinical variables when an intergroup comparison was made, it was observed that the LLLT (Group A) showed better results followed by Gengigel (Group B) (Table 2).

Another study compared the use of the 940 nm diode laser with scalpel surgery for the gingivectomy procedure, in terms of patient satisfaction. The postsurgical discomfort level was recorded using VAS. The researchers observed that the bleeding rate and pain level in the diode laser group were found to be less than in the scalpel group [16].

In this study, there was a marked difference in pain perception between the patients who received three different types of treatments, i.e., P<0.05. The LLLT group showed the least VAS scores followed by the Gengigel group, and, finally, the Hiora SG group.

Most of the studies assessing healing after gingivectomy have been done using the Haematoxylin and Eosin stain to observe the epithelization and the amount of connective tissue laid down, however, the quality of collagen laid down has not been assessed. So in this study, the Picrosirius red staining method was used to assess the quality of collagen laid down. As per our knowledge, Picrosirius red staining has not been used to assess healing after a gingivectomy.

The connective tissue is made up of collagen fiber bundles, which can be differentiated as thin and thick fibers. Type I and III collagens are predominant types in which type I collagen consists of thick and mature fibers and type III consists of thin and immature fibers [17-18].

Depending on the type, collagen exhibits a differential birefringence pattern, ranging from green-greenish yellow to orange-red, to red. Type I collagen fibers expressed an orange-red to red color, as they are made up of thick, mature, and closely packed fibrils presenting as an intense birefringence whereas type III collagen having a weak birefringence expressed a greenish-yellow to yellow color of the fibrils, as they were observed to be thin and immature.

The limitations of this study were the absence of a control group, which would have validated the study better. Moreover, the sample size was small and there were no studies available to compare the results of histological evaluation after six weeks post-gingivectomy with Picrosirius red.

## Conclusions

This study compared the efficacy of LLLT, Gengigel, and Hiora SG gel post-gingivectomy. Statistically significant results were observed in the LLLT group (Group A) pertaining to the GEI and VAS. Also, the histological evaluation showed more mature collagen fibers in the laser group. Gengigel performed better than Hiora SG gel both clinically and histologically, ranking next to the laser group. However, many more studies with a larger sample size have to be done to assert the benefits of LLLT and Gengigel, as was noticed in this study.
